# Association of preoperative electrocardiographic markers with sepsis in elderly patients after general surgery

**DOI:** 10.1186/s12872-023-03535-x

**Published:** 2023-10-04

**Authors:** WeiXian Xie, LiXia Wu, MeiXing Yang, HongLi Luo, Weichao Li, Heng Li

**Affiliations:** Department of Anesthesiology, The Sixth Affiliated Hospital of Guangzhou Medical University, Qingyuan People’s Hospital, Area B24, Yinquan Road, Xincheng District, Qingyuan City, Guangdong Province People’s Republic of China

**Keywords:** Postsurgical SS, P wave, PR interval, Preoperative ECG study, Predictor

## Abstract

**Background:**

Electrocardiographic markers, as surrogates for sympathetic excitotoxicity, are widely predictive of cardiovascular adverse events, but whether these markers can predict postsurgical sepsis (SS) is unclear.

**Methods:**

We retrospectively analyzed patients who underwent abdominal surgery from March 2013 to May 2023. We collected basic data, comorbidities, blood samples, echocardiology, electrocardiogram, and surgical data, as well as short-term outcome. The primary endpoints were postsurgical SS, in which logistic regression analyses can identify independent risk factors. The optimal cut-off value predictive postsurgical SS both P wave and PR interval were calculated in the receiver operating characteristic curve (ROC).

**Results:**

A total of 1988 subjects were analyzed, and the incidence of postsurgical SS was 3.8%. The mean age at enrollment was 68.6 ± 7.1 years, and 53.2% of the participants were men. In the ROC analysis, the areas under the curve (AUC) for P wave and PR interval predictive postsurgical SS were 0.615 (95%CI, 0.548–0.683; *p* = 0.001) and 0.618 (95%CI, 0.554–0.682; *p* = 0.001), respectively. The P wave and PR interval predicted postoperative sepsis with optimal discrimination of 103 and 157 ms, with a sensitivity of 0.744 and 0.419, and a specificity of 0.427 and 0.760. P-wave less than 103 ms or PR interval less than 157 ms associated with a 2.06 or 2.33 fold increase occurred risk postsurgical SS.

**Conclusions:**

Shorter P-wave and PR intervals were both independently associated with postsurgical SS. These preoperative electrophysiological markers could have potential useful for early recognition of postoperative SS.

## Introduction

Sepsis is a systemic chaos response to infection [1] that afflicts over 1,000,000 patients per year in the USA [2]. Severe sepsis accounts for 27% of ICU admissions in the UK [3] and affects over 30 million sufferers around the globe [4]. Perioperative sepsis is deadly and tricky, 40% of which is closely associated with cardiac arrest, and the mortality rate is over 70% for these cases [5]. Current guidelines are based on the belief that the cornerstone of effective sepsis treatment is early recognition, which is associated with improved outcomes [6]. There are limited tools for the early identification or screening of sepsis. Previous animal experiments suggested that colon puncture ligation (early sepsis) may induce gut-derived norepinephrine within 2 h [7, 8]. Sympathetic toxicity may precede the diagnosis of sepsis, whereas sympathetic stimulation can secondarily change various electrocardiographic markers [9], including P wave, PR interval, and QT duration [10, 11]. Sympathetic tachycardia and sympathetic-induced disorder with sodium, L-type Ca^2+^, and K^+^ current channels were associated with complicated infection [12]. The presence of Q-waves, left bundle branch block, QTc interval prolongation, J-waves, ST-segment changes, atrial fibrillation (AF), and high sharp T-waves can be detected on ECG [13, 14] in septic patients (diagnosed septic stage), representing the possibility of sympathetic-induced current channels disorder. Sympathetic toxicity (abnormal electrocardiogram markers) in the undiagnosed stage predicts sepsis is not fully understood.

The primary aim of this study was to explore the association of preoperative electrocardiographic markers (sympathetic toxicity) in the undiagnosed stage with postoperative sepsis.

## Methods

### Ethical statement and clinical trial registration

This study was authorized by the institutional ethics review committee before recruiting patients (Ethical Committee approval number: IRB-2022–080). This trial was registered at Chictr.org.cn (registration number: ChiCTR2200063917).

### Inclusion and exclusion criteria

In this study, we included in elderly patients over 60 years who underwent general surgery, including biliary, gastrointestinal, appendix, pancreatic, liver, and other surgeries. Some patients will be excluded with missed ultrasound electrocardiogram recording, electrocardiogram (ECG) recording, laboratory test results, medical records, and P wave or PR interval loss in ECG.

### The main outcomes and follow-up at the internal hospital

Sepsis is defined as life-threatening organ dysfunction caused by a dysregulated host response to infection. Postsurgical sepsis is defined as new-onset sepsis meeting the diagnostic criteria of “Sepsis-3” [1] after intra-abdominal surgery based on the recently updated Third International Consensus Definitions for Sepsis and Septic Shock (Sepsis-3). Cases were followed during their hospitalization, and the follow-up for clinical outcomes included 100% of the patients (1988 cases).

### Clinical data collected

All clinical data collected was derived from Qingyuan People’s Hospital Big Database. In the retrospective cohort study, we included the following covariates. Subject characteristics included age, sex, emergency surgery, and ASA III-IV. Comorbidities included hypertension, diabetes, COPD, a history of MI, coronary disease, valvular disease, pulmonary hypertension, and stroke. Surgical data included operative or anesthetic duration, laparoscopic surgery, conversion laparotomy, surgical indications, and blood transfusion. Echocardiography data included left or right atrial hypertrophy, left ventricular hypertrophy, pulmonary hypertension, cardiomegaly, and LVEF ≤ 50%. Laboratory tests included procalcitonin, WBC count, and C-reactive protein ≥ 40 mg/L. The outcomes were all-cause mortality, major cardiovascular events, ventricular tachycardia, moderate to severe anemia, malnutrition, incision infection, ARI or ARI requiring CRRT, and hospital stay.

### Electrocardiography data

All ECG data were digitally recorded and stored in a MedEx multilead ECG analysis system for MECG-300 type (Beijing Maddix Technology Co., Ltd., Beijing, China) and later automatically analyzed by ECG physicians using the processing software that came with the system. The assessment recording speed was set to more than 25 mm/s, and the sensitivity was set to 10 mm/mV. QT intervals were corrected for heart rate using Bazett’s formula (QTc = QT/RR^1/2^) [15]. The Tpeak-Tend interval (Tpe) is defined as the interval from the peak to the end of the T-wave in the V_2_ lead [16]. Tpe-Max is the longest Tpe among the 12 leads, and Tpe-Min is the shortest. Tpe dispersion is equal to the difference between Tpe-Max and Tpe-Min [17]. The P-wave duration is measured from the P-wave onset to its offset [18]. The maximum P-wave duration (P-wave-Max) is defined as the longest duration, and the minimum P-wave duration (P-wave-Min) is defined as the shortest duration on a standard 12-lead ECG. P-wave dispersion is equal to the difference between P-wave-Max and P-wave-Min [19]. The PR interval is defined as the duration from the onset of the P-wave to the initiation of the QRS segment [20].

### Statistical analyses

An analysis of postsurgical sepsis during hospitalization was conducted for this study. Discrete variables are presented as frequencies with their respective percentages, with continuous variables presented as the mean ± SD or median (IQR). For comparisons between two groups, continuous variables were analyzed by using Student’s t test or the Wilcoxon rank-sum test; if appropriate, categorical variables were analyzed with the Pearson chi-square test or Fisher’s exact test. Candidate variables with a *p value* < 0.05 in univariate analysis or the 2-group comparisons were entered into the models. Odds ratios of logistic regression analyses that can analyze independent markers for sepsis were calculated, with 95% CIs and associated *P* values. We analyzed the association between both presurgical electrocardiogram markers and sepsis in univariate and multivariate logistic regression models. Pre- and postsurgical clinical risk factors (including age, sex, ASA III-IV, emergency surgery, imipenem and vasoactive drugs used) entered the model.

The receiver operating characteristic curve (ROC) and area under the curve (AUC) were analyzed for P wave or PR interval. Subsequently, the optimal cut-off value predictive postsurgical SS both P wave and PR interval were calculated, with sensitivities and specificities relative to predictive postsurgical SS confirmed. Based on the optimal cut-off value of P-wave and PR intervals, we turned them into categorical variables, we again analyzed the association between both the optimal cut-off value and postsurgical SS in univariate and multivariate logistic regression models. Statistical significance was defined as a 2-sided *p-value* < 0.05. SPSS 25.0 software was used for all analyses.

## Results

**Fig. 1:**
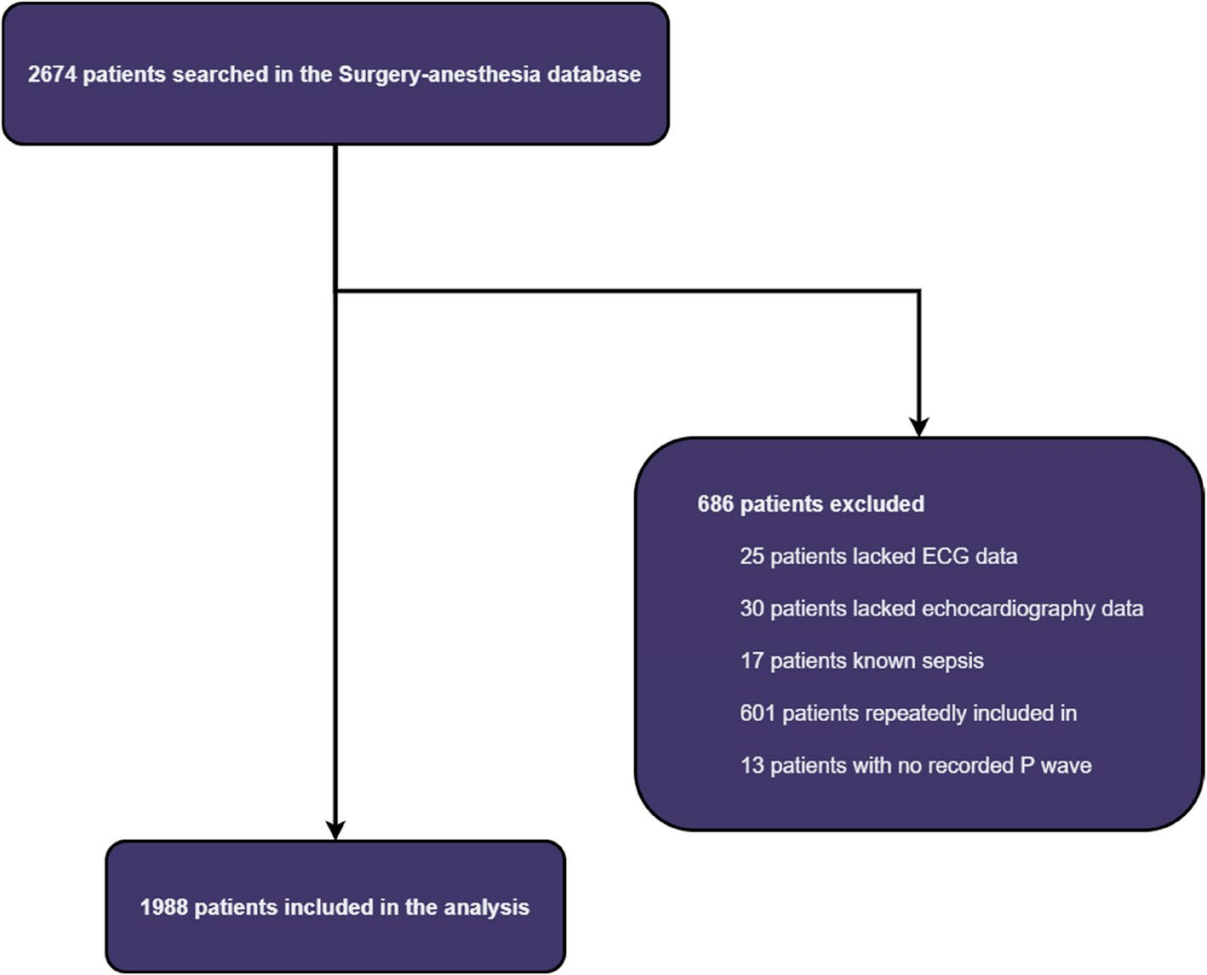
Flowchart of the research

A total of 2688 subjects were screened, and 1988 subjects who met the inclusion criteria were included in this study (Fig. 1). Table 1 shows the demographic, preoperative echocardiographic, laboratory, clinical, surgical, and prognostic characteristics of the included patients, as well as the preoperative electrocardiographic markers stratified by the occurrence of postsurgical SS.

**Table 1:** Clinical characteristics of the study cohort

	**Overall**	**No sepsis**	**sepsis**	***P* value**
	*N* = 1988	*N* = 1911	*N* = 77	
Covariates				
Subject characteristic				
Age, years	68.6 ± 7.1	68.5 ± 7.0	71.3 ± 9.4	0.001
Male (%)	1058 (53.2%)	1023 (53.6%)	35 (45.5%)	0.162
ASA III-IV, (%)	593 (29.8%)	539 (28.2%)	54 (70.1%)	< 0.001
Emergency surgery, (%)	563 (28.3%)	509 (26.6%)	54 (70.1%)	< 0.001
Comorbidities				
Hypertension, (%)	524 (26.4%)	503 (26.3%)	21 (27.3%)	0.853
Diabetes, (%)	216 (10.9%)	210 (11.0%)	6 (7.8%)	0.376
COPD, (%)	18 (0.9%)	16 (0.8%)	2 (2.6%)	0.11
A history of MI, (%)	5 (0.3%)	4 (0.2%)	1 (1.3%)	0.061
Coronary disease, (%)	224 (11.3%)	209 (10.9%)	15 (19.5%)	0.02
Valvular disease, (%)	21 (1.1%)	17 (0.9%)	4 (5.2%)	< 0.001
Pulmonary hypertension, (%)	82 (4.1%)	80 (4.2%)	2 (2.6%)	0.491
Stroke, (%)	70 (3.5%)	66 (3.5%)	4 (5.2%)	0.417
CHADS_2_	0.0 (0.0, 1.0)	0.0 (0.0, 1.0)	1.00(0.0, 2.0)	0.023
Charlson_index	2.0 (2.0, 4.0)	2.00(1.0, 4.0)	3.0 (2.0, 4.0)	0.446
Blood sample testing				
Procalcitonin, ng/mL	0.0 (0.0, 0.00)	0.0 (0.0, 0.0)	0.0 (0.0, 0.10)	< 0.001
White blood cell, × 10^9^/L	1.0 (1.0, 2.0)	1.0 (1.0, 1.0)	1.00(1.0, 10.0)	< 0.001
C-reactive protein ≥ 40, (%)	157 (7.9%)	144 (7.5%)	13 ( 16.9%)	0.003
Echocardiology data				
Left atria hypertrophy, (%)	183 (9.2%)	179 (9.4%)	4 (5.2%)	0.214
Right atria hypertrophy, (%)	48 (2.4%)	46 (2.4%)	2 (2.6%)	0.915
SWMA, (%)	37 (1.9%)	35 (1.8%)	2 (2.6%)	0.626
Left ventricle hypertrophy, (%)	16 (0.8%)	15 (0.8%)	1 (1.3%)	0.621
Right ventricle hypertrophy, (%)	3 (0.2%)	3 (0.2%)	0 (0.0%)	0.728
LVEF < 50%	15 (0.7%)	1(1.2%)	14 (0.7%)	0.574
Cardiomegaly, (%)	19 (1.0%)	17 (0.9%)	2 (2.6%)	0.131
Electrocardiogram data				
P-wave, ms	108.0 (102.0, 116.0)	108.0 (102.0, 116.0)	104.0 (94.0, 110.0)	< 0.001
PR interval, ms	151.42 ± 31.0	152.2 ± 30.0	133.5 ± 30.0	< 0.001
P-wave terminal potential,mm•s	0.0(0.0, 0.0)	0.0 (0.0, 0.0)	0.0 (0.0, 0.0)	0.579
P-wave area, ms•mv	9.1 (6.0, 11.4)	9.1 (6.1, 11.4)	8.6 (4.6, 11.2)	0.231
P-wave voltage	0.08 (0.06, 0.1)	0.08 (0.06, 0.1)	0.08 (0.05, 0.11)	0.755
P-wave Max, ms	108.0 (102.0, 116.0)	108.0 (102.0, 116.0)	104.0 (94.0, 110.0)	< 0.001
P-wave Min, ms	80.0 (70.0, 90.0)	80.0 (70.0, 90.0)	74.0 (62.0, 88.0)	0.014
P-wave dispersion, ms	28.0 (18.0, 38.0)	28.0 (18.0, 38.0)	24.0 (14.0, 34.0)	0.014
J-wave (%)	413 (20.8%)	397 ( 20.8%)	16 ( 20.8%)	0.999
ST change (%)	390 (19.6%)	366 (19.2%)	24 ( 31.2%)	0.009
QT, ms	400.0 (376.0, 426.0)	400.0 (376.0, 426.0)	374.0 (344.0, 424.0)	0.002
QTc, ms	435.0 (415.0, 458.0)	435.0 (415.0, 458.0)	447.0 (427.0, 468.0)	0.004
QRS, ms	88.0 (82.0, 94.0)	88.0 (82.0, 94.0)	88.00 (82.0, 94.0)	0.825
Tpe, ms	102.0 (86.0, 118.0)	102.0 (88.0, 118.0)	104.00 (82.0, 118.0)	0.675
Tpe/QT	0.26 (0.22, 0.30)	0.26 (0.22, 0.30)	0.25 (0.22, 0.30)	0.526
Tpe/QTc	0.24 (0.20, 0.27)	0.24 (0.20, 0.27)	0.23 (0.18, 0.26)	0.164
Surgical data				
Laparoscope (%)	1215 (61.1%)	1179 ( 61.7%)	36 ( 46.8%)	0.008
Conversion laparotomy (%)	30 ( 1.5%)	24 ( 1.3%)	6 ( 7.8%)	< 0.001
Gastrointestinal perforation (%)	241 (12.1%)	206 (10.8%)	35 (45.5%)	< 0.001
Obstruction (%)	452 (22.7%)	423 (22.1%)	29 (37.7%)	0.001
Intestinal tumor (%)	1211 (60.9%)	1178 (61.6%)	33 (42.9%)	0.001
Ulcer (%)	415 (20.9%)	402 ( 21.0%)	13 (16.9%)	0.379
Biliary stones (%)	452(22.7%)	431( 22.5%)	21(27.2%)	0.245
Blood transfusion, (%)	289 (14.5%)	280 (14.7%)	9 (11.7%)	0.469
Operation duration, h	2.50 (1.30, 4.17)	2.50 (1.30, 4.20)	2.40 (1.40, 3.50)	0.404
Anesthesia duration, h	3.40 (2.15, 5.10)	3.40 (2.15, 5.15)	3.50 (2.40, 4.50)	0.519
Prognosis				
Imipenem used, (%)	23 (1.2%)	10 (0.5)	13 (16.9)	< 0.001
Vasoactive drugs used, (%)	67 (3.4%)	38 (2.0%)	29 (37.7)	< 0.001
Atrial fibrillation (%)	23 (1.2)	21 (1.1)	2 (2.6)	0.228
Ventricular tachycardias	50 ( 2.5)	40 ( 2.1)	10 ( 13.0)	< 0.001
Major cardiovascular events	62 ( 3.1)	47 ( 2.5)	15 ( 19.5)	< 0.001
Undernutrition (%)	19 ( 1.0)	16 ( 0.8)	3 (3.9)	0.007
Acute renal injury (%)	1 (3.4)	1 (3.6)	0 (0.0)	0.847
CRRT (%)	28 (1.4)	20 (1.0)	8 (10.4)	< 0.001
Severe anemia (%)	68 (3.4)	62 (3.2)	6 (7.8)	0.031
Moderate anemia (%)	146 (7.3)	136 (7.1)	10 (13.0)	0.053
Incision_infection (%)	29 (1.5)	23 (1.2)	6 (7.8)	< 0.001
Hospital stay, day	16.0 (11.0, 22.0)	16.0 (11.0, 22.0)	20.0 (14.0, 28.0)	< 0.001
All-cause mortality, (%)	30 (1.5%)	17 (0.9%)	13 (16.9%)	< 0.001

Continuous data are presented as mean ± SD or median (interquartile range); Discrete data are presented as frequencies with their respective percentages. *P* value of Student’s t, Fisher’s exact,or the Wilcoxon rank-sum test between groups of patients with and without sepsis

### Incidence and cohort characteristics of postsurgical SS

The incidence of postsurgical SS was 3.8%, which is most common in patients with gastrointestinal perforation, obstruction, intestinal tumors, and biliary stones. Postsurgical SS occurred in 77 patients, who had 16 cases (16.9% vs. 0.9%, *p* < 0.001) of hospitalization death. The occurrence of SS significantly prolonged the hospital stay [20.0 (14.0, 28.0) vs. 16.0 (11.0, 22.0), *p* < 0.001] (Table 1).

### ROC analyses for the P wave and PR interval predictive postsurgical SS

**Fig. 2:**
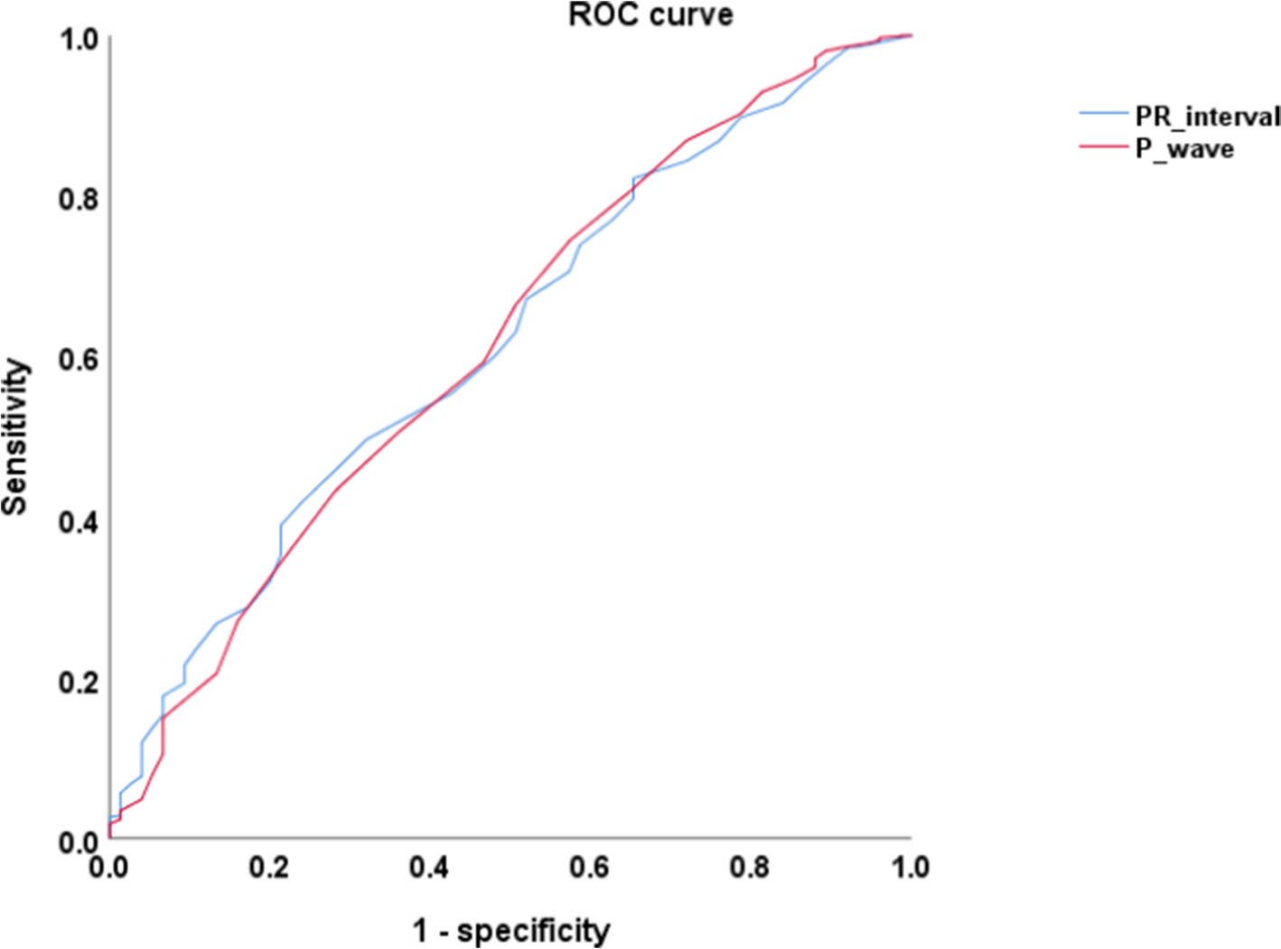
ROC analysis of P wave and PR interval. The areas under the curve (AUC) for P wave and PR interval were 0.615 (95%CI, 0.548–0.683; *p* = 0.001) and 0.618 (95%CI, 0.554–0.682; *p* = 0.001), respectively. The P wave or PR interval predicted postoperative sepsis with a Yuden index of 0.171 or 0.179, a sensitivity of 0.744 or 0.419, a specificity of 0.427 or 0.760, and optimal discrimination of 103 or 157 ms

Figure 2 showed that the areas under the curve (AUC) for P wave and PR interval were 0.615 (95%CI, 0.548–0.683; *p* = 0.001) and 0.618 (95%CI, 0.554–0.682; *p* = 0.001), respectively. The P wave and PR interval predicted postoperative sepsis with optimal discrimination of 103 and 157 ms, with a sensitivity of 0.744 and 0.419, and a specificity of 0.427 and 0.760.

### Univariate and multivariable analyses for the association of P-waves < 103 ms or PR intervals < 157 ms with postsurgical SS

Based on the optimal discrimination of P-wave and PR intervals, we turned them into categorical variables. P-wave < 103 ms or PR interval < 157 ms less than 157 ms associated with a 2.06 (adjusted OR, 2.06; 95% CI, 1.27 -3.30; *p* = 0.003) or 2.33 (adjusted OR, 2.33; 95% CI, 1.33 -4.10; *p* = 0.003) fold increase occurred risk postsurgical SS in multivariable analysis (Table 2).

**Table 2:** Univariate and multivariate logistics regression analyses with possible risk factors predictive postsurgical SS

Covariates	Univariate analysis	^*^P value	Multivariate analysis	^#^*P* value
	Unadjusted HR 95%CI		Adjusted HR 95%CI	
Age	1.04(1.01–1.07)	0.017	1.03(1.00 -1.06)	0.02
Male	1.35(0.81–2.23)	0.247		
ASA III-IV	4.99(2.93–8.50)	< 0.001	4.17(2.45–7.10)	< 0.001
Emergency surgery	6.79(3.90–11.84)	< 0.001	3.06 (1.71–5.47)	< 0.001
Hypertension	1.05 (0.63–1.75)	0.853		
Diabetes	0.69(0.29–1.59)	0.380		
COPD	3.16(0.71–13.99)	0.130		
A history of MI	7.75(0.85–70.32)	0.069		
Coronary disease	1.70(0.87–3.31)	0.118		
Valvular disease	7.61(2.48–23.31)	< 0.001	11.55(3.06–43.66)	0.59
Pulmonary hypertension	0.61(0.15–2.53)	0.496		
Stroke	1.53(0.54–4.32)	0.420		
CHADS_2_	1.40(1.10–1.79)	0.006	1.07(0.79–1.46)	0.64
Charlson_index	1.03(0.94–1.12)	0.568		
Procalcitonin	1.03(1.01–1.05)	0.002	1.58(0.73–3.40)	0.24
White blood cell	1.08(1.05–1.12)	< 0.001	1.05(1.01–1.09)	0.02
C-reactive protein ≥ 40	2.00(0.97–4.13)	0.061	1.07(0.51–2.22)	0.87
Left atria hypertrophy	0.53(0.19–1.47)	0.222		
Right atria hypertrophy	1.08(0.26–4.54)	0.915		
SWMA	1.43(0.34–6.05)	0.628		
Left ventricle hypertrophy	1.66(0.22–12.76)	0.625		
Right ventricle hypertrophy	0.00(0.00–15.59)	0.999		
LVEF < 50%	1.78(0.23–13.73)	0.579		
Cardiomegaly	2.97(0.67–13.09)	0.150		
P-wave, ms	1.31(1.30–1.64)	0.007	1.42 (1.09–1.53)	0.006
PR interval	1.13(1.08–1.18)	< 0.001	1.12 (1.07–1.18)	< 0.001
P-wave < 103 ms	2.64(1.67–4.20)	< 0.001	2.06(1.27–3.30)	0.003
PR interval < 157 ms	2.35(1.38–4.00)	0.002	2.33(1.33–4.10)	0.003
P-wave terminal potential	0.00()0.00–67.93	0.888		
P-wave area	0.97(0.95–0.99)	0.007	0.95(0.90–1.00)	0.05
P-wave voltage	0.21(0.00–65.93)	0.598		
P-wave Max	1.01(1.01–1.02)	0.001	1.03 (1.02–1.05)	0.01
P-wave Min	1.01(1.01–1.03)	0.204		
P-wave dispersion	0.98(0.97–1.00)	0.034		
J-wave	1.28(0.68–2.38)	0.448		
ST change	0.52(0.32–0.85)	0.009	0.80(0.47–1.35)	0.40
QT	1.01(1.00–1.01)	0.004	1.00(0.99–1.00)	0.16
QTc	1.01(1.00–1.02)	0.011	1.01(1.00–1.01)	0.09
QRS	1.01(1.00–1.02)	0.046		
Tpe	1.00(0.99–1.01)	0.611		
Tpe/QT	5.63(0.49–64.17)	0.164		
Tpe/QTc	0.27(0.01–10.37)	0.484		
Laparoscope	0.53(0.32–0.88)	0.014		
Conversion laparotomy	6.55(2.42–17.72)	< 0.001	3.86(1.30–11.41)	0.02
Gastrointestinal perforation	6.90(4.31–11.05)	< 0.001	3.79(2.17–6.63)	< 0.001
Obstruction	2.13(1.32–3.41)	0.044	1.61(0.96–2.69)	0.07
Tumor	2.44(1.46–4.08)	0.001	0.77(0.46–1.28)	0.31
Ulcer	0.76(0.42–1.40)	0.381		
Blood transfusion	0.77(0.38–1.56)	0.471		
Operation duration	0.95(0.92–0.97)	< 0.001	0.97(0.95–1.00)	0.07
Anesthesia duration	1.00(0.89–1.12)	0.992		
Imipenem used	2.04(0.62–6.75)	0.242		
Vasoactive drugs used	0.63(0.15–2.62)	0.527		
Atrial fibrillation	5.57(2.51–12.36)	< 0.001	3.44(1.37–8.63)	0.01
Ventricular tachycardias	7.66(3.54–16.55)	< 0.001	1.41(0.53 -3.73)	0.491
Major cardiovascular events	9.95(5.08–19.51)	< 0.001	5.84(2.80–12.16)	< 0.001
Undernutrition	4.80(1.37–16.83)	0.014	3.69(0.93–14.60)	0.06
Acute renal injury	8.00(2.55–25.12)	< 0.001	4.66(1.29–16.79)	0.02
CRRT	10.96(4.66–25.75)	< 0.001	6.28(2.23–17.70)	< 0.001
Severe anemia	2.52(1.05–6.02)	0.038		
Moderate anemia	1.95(0.98–3.87)	0.057		
Incision_infection	6.93(2.74–17.56)	< 0.001	6.01(2.05–17.61)	< 0.001
Hospital stay	1.03(1.01–1.04)	0.001	1.03(1.02–1.05)	0.001
All-cause mortality	21.22(9.61–46.85)	< 0.001	10.72(4.39–26.21)	< 0.001

^*^The event rates were unadjusted

^#^Adjusted for age, sex, ASA III-IV, emergency surgery, imipenem and vasoactive drugs used listed in Table 1

Table 2 also showed results for univariate and multivariate Logistics Regression Analysis about postoperative SS. Age, ASA III-IV, emergency surgery, CHA_2_DS_2_VASc score, white blood cell, gastrointestinal perforation, conversion laparotomy,atrial fibrillation, major cardiovascular events, CRRT, acute renal injury, incision infection, hospital stay, and all-cause mortality were independent risk factors for postoperative SS.

## Discussion

We found that (1) abnormal preoperative ECG parameters preceded postsurgical SS. (2) A decreased P-wave and PR interval were both independently associated with postsurgical SS. As a dynamic process, sepsis can turn into conditions of varied severity [21, 22]. There is an inflammatory response that is determined by the pathogenic agent and the host (genetic characteristics and coexisting illnesses) in patients with sepsis, with differential responses at the local, regional, and systemic levels. The infection source not only extends to the infected tissue or organ but also induces either secondary peritonitis or combined peritonitis in complicated intra-abdominal infections (IAIs). Sympathetic excitotoxicity and released plasma norepinephrine levels were observed in peritonitis patients and experimental peritonitis animals [23]. An increased sympathetic tone is thought to be compensatory in the initial infectious stage, but its continuous activation may become pathological. Persistent tachycardia secondary to catecholamine is common [24]. In later stages of severe peritonitis, β-adrenoceptor stimulation from maintenance of the sympathetic tone results in various ionic currents being markedly depressed in atrial and ventricular myocytes. The use of β-blockers in patients with sepsis with persistent tachycardia was associated with significantly improved short-term outcomes [25]. Alternative mechanisms are associated with reductions of L-type calcium currents and sodium ion currents due to pro-inflammatory cytokines induced by serious peritonitis.

Decreased P-waves were independently associated with postsurgical SS. There are few previous studies considering the possibility of using electrocardiography as a screening tool for sepsis [26, 27], while numerous studies have focused on using electrocardiographic diagnosis for septic atrial or ventricular tachyarrhythmias. The P-wave represents the time required for a sinus impulse to propagate from the sinus node to the entire atrium [28]. A decreased P-wave duration correlates with a fast conduction velocity within the atria [29]. Angelo et al. found that a 28% reduction in action potential duration 90 (APD_90_) yielded a 22% reduction in P-wave duration [30]. The underlying mechanisms include a shortened action potential duration (APD) in atrial cells from LPS-induced septic animals associated with a reduced L-type Ca^2+^ current and sodium channels and an increased delayed rectifier K^+^ current [31]. Sympathetic tachycardia is widely prevalent in critical illnesses, including sepsis, trauma, burns, and cardiac arrest [32]. Isoproterenol infusion significantly shortened the P-wave duration for healthy volunteers in an autonomic stimulation test [33]. Under physiological and pathological conditions, the PR interval diminishes with increasing HR [34]. Although associations between cardiac conduction and sepsis in humans have not been identified so far, there are verified associations between a decreased PR interval and sepsis in animal models [35, 36]. This is consistent with the results of this study that preoperative shorter PR intervals were strongly predictive of postsurgery SS. Gianfranco Piccirillo et al. also suggested a possible ANS influence on the PR interval [37]. In summary, our results confirm the potential value of electrophysiological markers for predicting sepsis in the setting of general surgery.

Elderly patients with complicated IAIs present to physicians with fewer signs of peritonitis and have little inflammatory response [38]. In many developing countries worldwide, there is still a significant unacceptable delay in admitting older patients to the hospital [39]. Elderly patients may be preseptic in the preoperative stage, but current screening methods do not provide early recognition. The Sepsis-3 criterion requiring already present organ failure has a deficit in its predictive potential and may obstruct early recognition and treatment of sepsis [1]. Quick SOFA (qSOFA) does not diagnose sepsis, likely discriminating against low- or high-risk sepsis inside and outside critical care units [40]. Early warning scores are based on abdominal signs and symptoms, which are often absent in the elderly [41]. A new method of early septic screening is essential to improve patient outcomes. Our study showed that early changes in ion channel currents associated with sepsis could be identified on the ECG, and anesthesia or critical illness practitioners are able to grasp the characteristics of the electrocardiogram.

### Study limitations

Regarding this study’s limitations, the limited population, single-center origin, and lack of randomness should be considered. In addition, information about any unacceptable delay before arriving at the hospital was not included, and this information is necessary to help develop effective screening based on data in the real world. The types of bacterial infections were not clearly identified. Racial heterogeneity can lead to biased data, but negative trends in these markers are dominant.

## Conclusion

Electrocardiographic markers as a surrogate for sympathetic excitotoxicity have an independent association with postsurgical SS. Preoperative electrocardiographic markers have potential predictive value for complicated intra-abdominal infections.

## Data Availability

The data can be obtained from the corresponding authors upon reasonable request.

## Abbreviations

LPS: Lipopolysaccharide
SS: Sepsis
SWMA: Segmental wall motion abnormality
COPD: Chronic obstructive pulmonary disease
MI: Myocardial infarction
ASA: American Society of Anesthesiologists
CRRT: Continuous renal replacement therapy
ARI: Acute renal injury
WBC: White blood cell
LVEF: Left ventricular ejection fraction
